# Compliance to Oseltamivir among Two Populations in Oxfordshire, United Kingdom Affected by Influenza A(H1N1)pdm09, November 2009 – A Waste Water Epidemiology Study

**DOI:** 10.1371/journal.pone.0060221

**Published:** 2013-04-15

**Authors:** Andrew C. Singer, Josef D. Järhult, Roman Grabic, Ghazanfar A. Khan, Ganna Fedorova, Jerker Fick, Richard H. Lindberg, Michael J. Bowes, Björn Olsen, Hanna Söderström

**Affiliations:** 1 NERC – Centre for Ecology and Hydrology, Wallingford, United Kingdom; 2 Section of Infectious Diseases, Department of Medical Sciences, Uppsala University, Uppsala, Sweden; 3 Department of Chemistry, Umeå University, Umeå, Sweden; 4 University of South Bohemia in České Budějovice, Faculty of Fisheries and Protection of Waters, South Bohemian Research Center of Aquaculture and Biodiversity of Hydrocenoses, Vodnany, Czech Republic; 5 Section for Zoonotic Ecology and Epidemiology, School of Natural Sciences, Linnaeus University, Kalmar, Sweden; Nanyang Technical University, United States of America

## Abstract

Antiviral provision remains the focus of many pandemic preparedness plans, however, there is considerable uncertainty regarding antiviral compliance rates. Here we employ a waste water epidemiology approach to estimate oseltamivir (Tamiflu®) compliance. Oseltamivir carboxylate (oseltamivir's active metabolite) was recovered from two waste water treatment plant (WWTP) catchments within the United Kingdom at the peak of the autumnal wave of the 2009 Influenza A (H1N1)pdm09 pandemic. Predictions of oseltamivir consumption from detected levels were compared with two sources of national government statistics to derive compliance rates. Scenario and sensitivity analysis indicated between 3–4 and 120–154 people were using oseltamivir during the study period in the two WWTP catchments and a compliance rate between 45–60%. With approximately half the collected antivirals going unused, there is a clear need to alter public health messages to improve compliance. We argue that a near real-time understanding of drug compliance at the scale of the waste water treatment plant (hundreds to millions of people) can potentially help public health messages become more timely, targeted, and demographically sensitive, while potentially leading to less mis- and un-used antiviral, less wastage and ultimately a more robust and efficacious pandemic preparedness plan.

## Introduction

An influenza pandemic is regarded as one of the most significant civil emergency risks with major global human health consequences and the potential to cause significant social and economic damage and disruption [1], [2]. One of the few options for alleviating the human health burden from an influenza pandemic is the use of pharmaceuticals such as antivirals. Vaccine provision and non-pharmaceutical measures (e.g., closing schools and/or borders, hand-washing) represent two other widely used infection mitigation approaches employed in national preparedness plans. Numerous countries world-wide distributed courses of antivirals for prophylaxis and treatment of influenza-like illness (ILI) during the 2009 Influenza A(H1N1)pdm09 pandemic, with the majority of the antiviral stockpiles consisting of oseltamivir (Tamiflu®) [3].

The United Kingdom Health Protection Agency (HPA) QSurveillance National Syndromic Surveillance System monitored a range of clinical and syndromic indicators that were indicative of influenza activity. This system was established when antiviral drugs were deployed during the pandemic in the U.K. The National Pandemic Flu Service (NPFS) was responsible for the provision of information, syndromic diagnosis, and prescription and dispensing of antiviral courses in the UK.

Compliance rates to the prescribed antiviral course (i.e., one dose of Tamiflu per day for prophylaxis and two doses per day for treatment) was first reported in the U.K. three months after the outset of the pandemic [4]–[8]. Compliance was highly varied, ranging between 48 to 97%, with a narrow age range of study participants, <14 yrs of age. To our knowledge, compliance rates for the period of the second, autumnal wave of the 2009 influenza pandemic in the U.K. was not collected, nor was there any significant body of research to draw upon for predicting compliance in >14 yr olds at any point during the 2009 pandemic. This knowledge gap greatly hinders the ability of government and public health planners to proactively address the problem of compliance and the issues it generates.

Poor compliance drains resources by diverting limited antiviral stocks from those who may need it most. Mis- and un-used antivirals can lead to the hastening of antiviral resistance in cases where influenza-infected people do not comply with the prescribed course and dosing regimen. The provision of antivirals that remain unused also represents a significant financial cost to governments [9]. Antiviral non-compliance can also influence the success of inter-related public health plans, such as combating secondary bacterial infections in influenza cases. The provision of antivirals is expected to decrease the need for antibiotics by an estimated ∼50% owing to a reported decline in secondary infections in antiviral users [3], [10], [11]. For these reasons, the pandemic influenza medical response and the national pandemic preparedness plan will remain unnecessarily vulnerable without greater certainty with respect to human behaviour – more specifically, antiviral compliance.

Oseltamivir carboxylate (OC; oseltamiviŕs active metabolite) is frequently demonstrated to be a conservative chemical in waste- and fresh-water systems [12]–[20], and as such, represents an ideal tracer for the waste water forensic epidemiology approach [21]–[23]. In this epidemiological study we evaluated the load of OC in waste water as an unbiased measure of Tamiflu consumption during an influenza pandemic. The OC levels in influent of two waste water treatment plants (WWTPs) located at Benson (51.61562, −1.10945) and Oxford (51.71384, −1.21545), in Oxfordshire, England, were measured during the peak of the 2009 Influenza A(H1N1)pdm09 pandemic [24]. Measured OC was compared with two complementary sources of national government statistics to assess compliance rates. It is proposed that an empirically-derived estimate of compliance, recorded in near-real time, can help to inform and prioritise public health messages at the spatial resolution of the WWTP catchment, which can range from a population of a few thousand in rural areas to over a 1 million in highly urban areas. We argue that this granular understanding of non-compliance can help public health messages become more targeted and efficacious, leading to less mis- and un-used antivirals, cost savings and a more robust preparedness plan.

## Methods

### Waste Water Sampling

An urban and a rural WWTP were chosen for this study to reflect potentially different pharmaceutical use patterns in the two catchment populations. The rural WWTP at Benson England, serves a population of 6,230 people with a consented dry weather flow of 2,517 m^3^/d and an annual average dry weather flow (DWF) of 1,368 m^3^/d. The Benson WWTP has a hydraulic retention time of 7–8 h at dry weather flow and consists of trickling filters as the main biological treatment step. The urban WWTP at Oxford serves a population of 208,000 with a consented dry weather flow of 50,965 m^3^/d and an annual mean DWF of 38,000 m^3^/d. The Oxford WWTP has a hydraulic retention time of 15–18 h, and utilizes activated sludge as the main biological treatment step. Both WWTPs have primary and secondary sedimentation steps. The Oxford and Benson sewer systems receive flow from a number of pumping stations either running in series to the site along the sewer network or in parallel from sub-catchments.

Thames Water Utilities Limited provided access to both WWTP; all necessary permits were obtained for described field studies, including the Thames Water Operational Safety Authorization (TWOSA). Each WWTP was sampled using an automated sampler scheduled to recover a time-proportional sample (approximately 750 mL) of influent every hour for 24 hours. Sampling commenced at 15∶00 on Nov 10^th^, with the last sample taken at 14∶00 on Nov 11^th^ 2009. At its completion, samples were stored, in triplicate, in 50-mL borosilicate glass vials with PTFE-lined caps at −80°C. Samples were shipped frozen to Umeå University, Sweden, where they were stored at −20°C until analysis.

**Figure 1:**
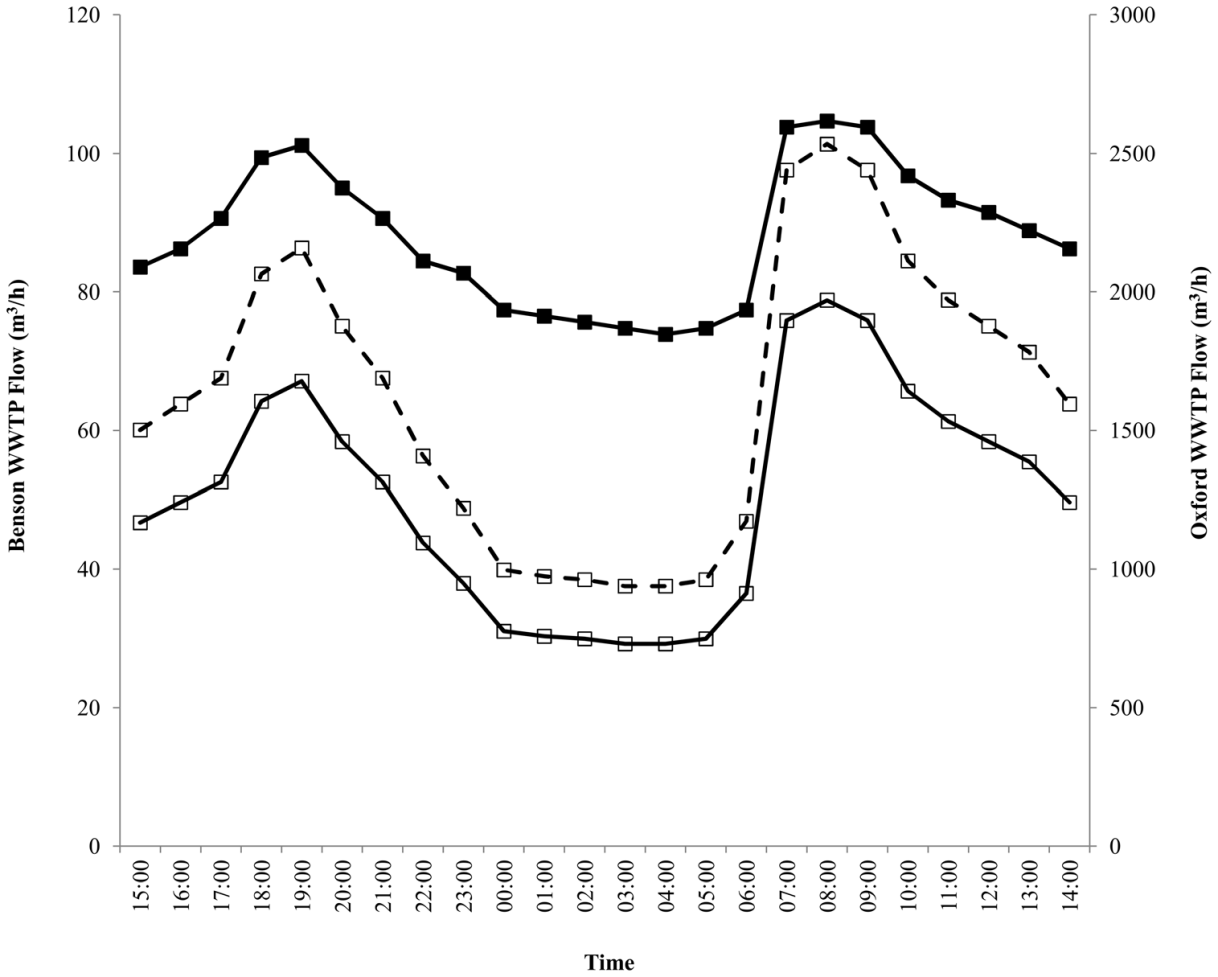
Waste Water Treatment Plant Flow. Hourly flow (m^3^/h) from Benson (open) and Oxford (shaded) WWTP on sampling days 11 November 2009 (solid line) and 10 May 2011 (dashed line).

OC was converted to mass loading using hourly WWTP flows for the sampling period (Figure 1). Flows were determined in consultation with Thames Water, the WWTP operator. Flows at both WWTPs peaked between 07∶00 to 9∶00 and again from 18∶00 to 19∶00. An additional 24-h sampling was initiated at 10∶00 on 15 May, 2011 from only the Benson WWTP effluent for the purpose of confirming the background concentration of antiviral during the inter-pandemic period, which officially began on 10 August, 2010 [25].

### Environmental Conditions

Precipitation on 11 November, 2009 (3.3 mm) was approximately 1.0 mm below the monthly average for November (4.25 mm) [26]. The 24-hour mean flow for Benson WWTP was 1,210 m^3^/d, 13% below the average annual dry weather flow. Oxford WWTP's daily flow was 52,828 m^3^/d, 28% higher than the annual average dry weather flow for the same time period. No precipitation occurred within the previous 48 h of the May 11, 2011 sampling point of the Benson WWTP influent where the mean 24-h flow was 1556 m^3^/d. The temperature during the sampling period ranged from 0.7 to 10.5°C and 6 to 19.6°C over the November 11, 2009 and May 11, 2011 sampling periods, respectively [26].

### Measurement of OC in Waste Water

An on-line solid phase liquid extraction/liquid chromatography-tandem mass-spectrometry (SPE/LC-MS/MS) system was used to measure the OC levels in the samples collected at Benson and Oxford WWTPs in southern Oxfordshire, England. The SPE/LC-MS/MS system used has been evaluated and described in details previously [27]. Briefly, 1 mL of 5 mL pre-filtered (0.45 µm pore size) sample was analyzed by the SPE/LC-MS/MS system. The samples were quantified using a deuterated OC internal standard, with six calibration points. The limit of quantification (LOQ) was 2 ng/L.

### Predicting Tamiflu Consumption

Two methods were used for predicting Tamiflu consumption within the two WWTP catchments, based on data from the National Pandemic Flu Service (NPFS) [24] and the HPA/QSurveillance National Syndromic Surveillance System (HPA) [28]. The NPFS recorded the collection of 1,079,179 courses of antiviral treatment in England between the launch of NPFS in July 2009 to February 2010, when it ceased operation, equating to ∼2% of the population. Using these values, an estimated 132 courses of antivirals were dispensed in the Benson WWTP catchment and 4,401 in the Oxford WWTP catchment over the same period of time. Approximately 66,218 courses of antiviral (6% of all antivirals dispensed) were dispensed in Week 43 (3 weeks prior to the WWTP sampling), the national peak for the autumnal wave of the 2009 influenza pandemic [24], equating to approximately 0.13% of the population of England receiving antiviral. An exact amount of antivirals dispensed for Week 46 (the week of the study) was not available, however, it is estimated that there was less than a 10% decline in antiviral dispensing by Week 46, thereby making any antiviral allocation differences between Week 43 and 46 negligible (see Figure 15 in ref [24] for antiviral collections during the pandemic). These predictions translate into: 8 and 270 courses of antiviral collected within the Benson and Oxford WWTPs catchment during the week of sampling (10 November, 2009). The following compliance scenarios were examined: 40, 45, 50, 55, 60, 70 or 100% compliance (i.e. 40–100% of those collecting Tamiflu would use it, as directed). The standard dosing regime was assumed: 0.075 g per dose, consumed twice per day (0.150 g/d).

The HPA dataset reports 54.2 people per 100,000 with ILI in the Oxfordshire PCT during Week 46 (inclusive of both the Oxford and Benson WWTP catchments). This prediction translates to 3·4 and 112·7 cases of ILI in the Benson and Oxford catchments, respectively. In addition to the seven compliance scenarios previously mentioned for the NPFS dataset, an additional scenario was needed for the HPA dataset: 50 or 100% of the cases of ILI were prescribed antiviral. This additional scenario was examined because only ∼50% of ILI cases are clinically-diagnosed with influenza. The antiviral prescription rate for ILI might be expected to more closely approximate 100% than the more clinically-accurate 50% prescription rate, as syndromic diagnosis prevails during a pandemic, with clinical diagnosis more the exception than the rule.

### Predicted OC Concentrations in Waste wsater

The projected concentration of OC in the waste water (ng/L) was calculated using Equation 1, 
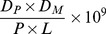
(1)where the product of the population of each catchment (*P*; Benson  = 6230 and Oxford  = 208000) and the volume of waste water per person (*L*) (230 L) was divided into the product of population predicted to consume Tamiflu (*D_p_*) and the mass equivalent of OC consumed per day in grams (*D_M_*; 0·15 g/d is the defined daily dose (DDD) for Tamiflu for treatment purposes).

An additional scenario that assumes either 80 or 100% of the Tamiflu dose was recovered as OC in WWTP influent was employed when predicting concentrations of OC in waste water or back-calculating from waste water to Tamiflu users. This scenario was employed as 100% of the Tamiflu dose is excreted into the waste water, with up to 20% in the form of the prodrug oseltamivir. However, the prodrug can potentially transform in the waste water to the active antiviral, OC, leading to a theoretical maximum of 100% of consumed Tamfilu recovered as OC [29]. This scenario was necessary because the parent prodrug was not monitored in the waste water. If the ratio of parent compound to active metabolite was found to be approximating 1∶4, a default scenario of 80% would be sufficient.

## Results

### Measured Antiviral Concentrations or Load in Waste water

**Figure 2:**
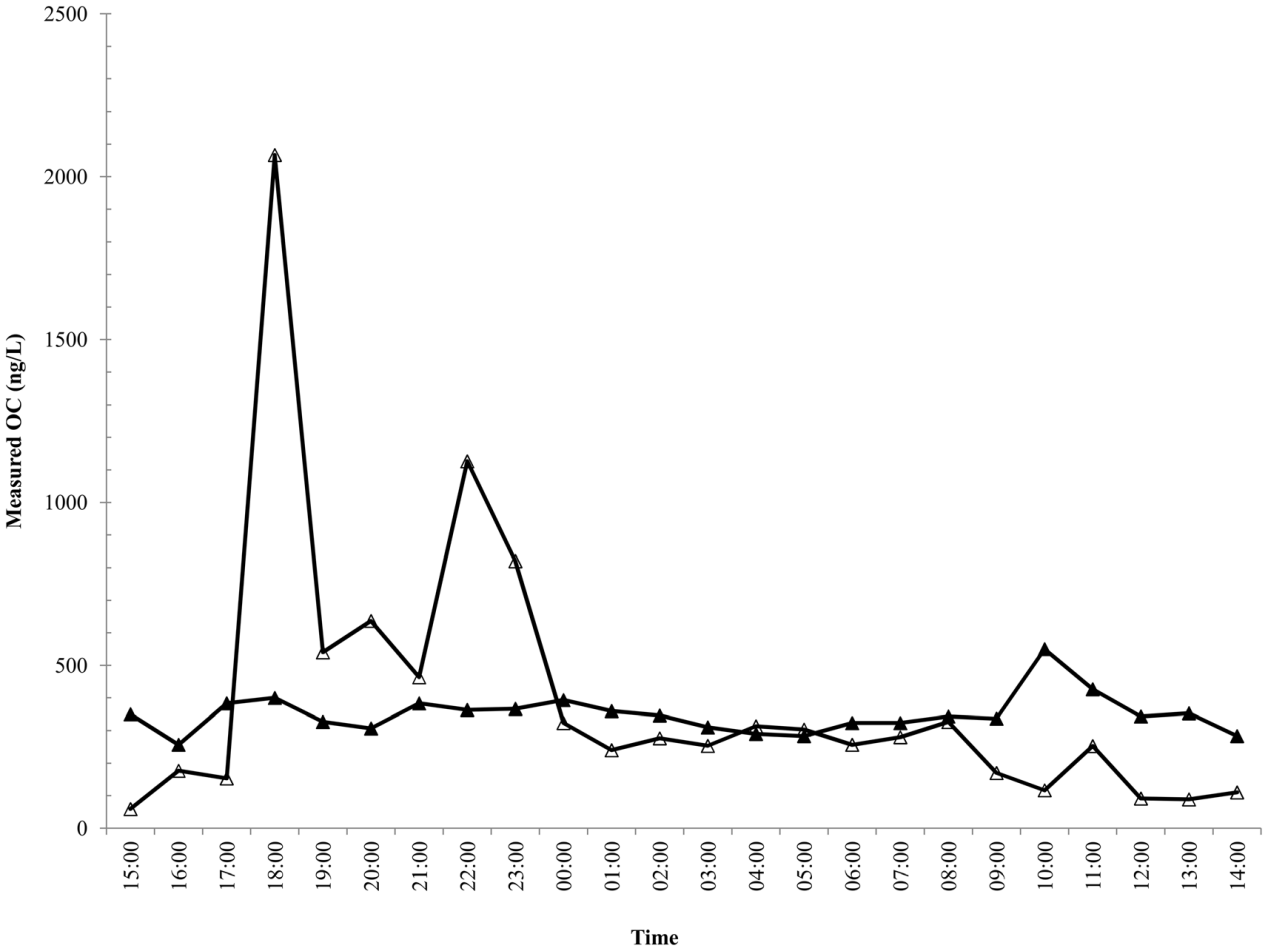
Oseltamivir Carboxylate Concentration in Waste Water. Hourly time-proportional influent concentration of OC (ng/L) in Oxford WWTP (shaded) and Benson WWTP (open).

**Figure 3:**
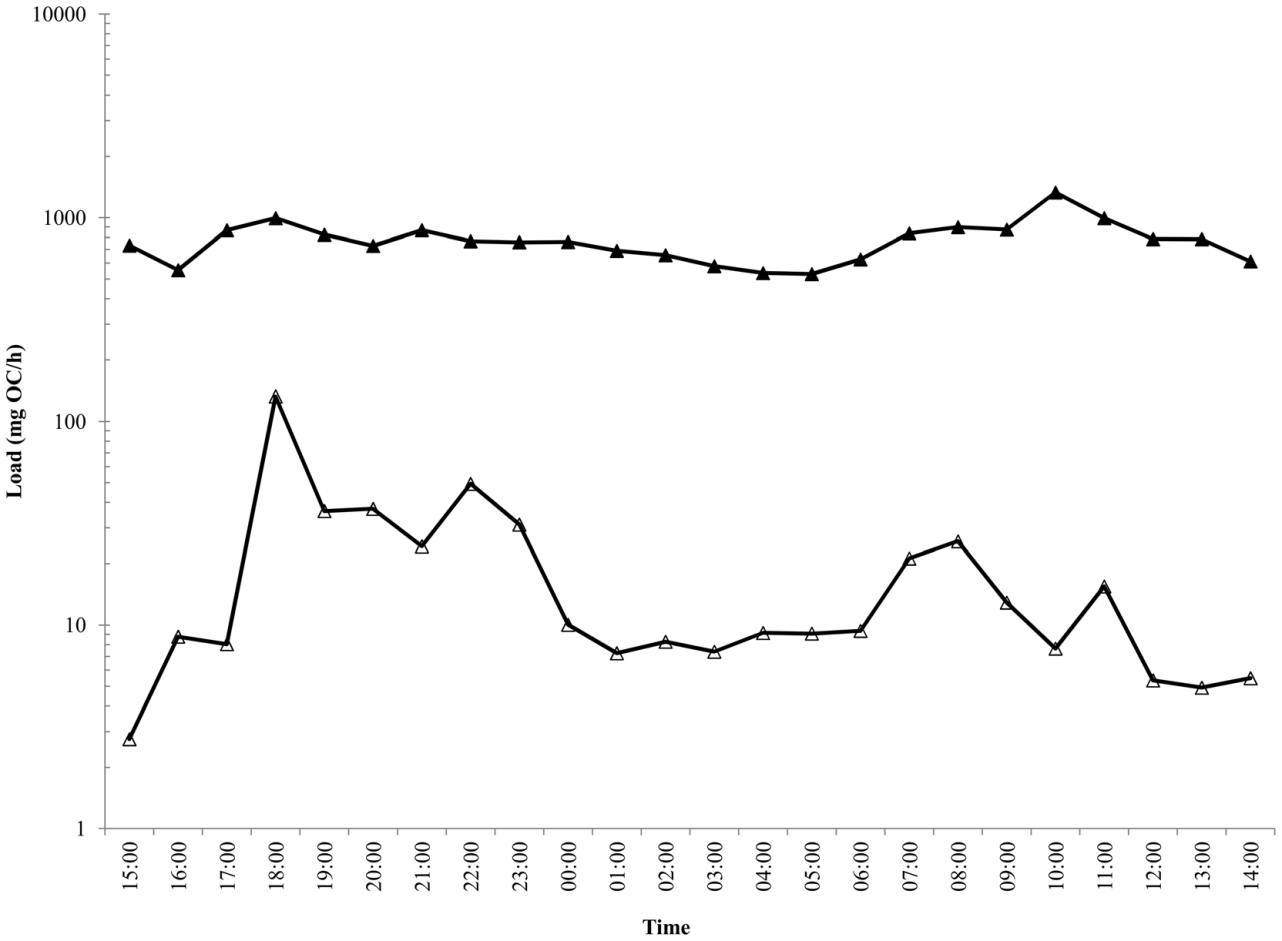
Oseltamivir Carboxylate Load in Waste Water. Calculated hourly influent load of OC (mg/h) for Oxford WWTP (closed) and Benson WWTP (open).

The concentration of OC in the influent of Benson WWTP ranged from 59 to 2,070 ng/L, with a mean of 394±435 ng/L (Figure 2). The average load for the 24 h period was 490±26.9 mg/h (Figure 3). The concentration of OC in the influent of Oxford WWTP ranged from 257 to 550 ng/L, with a mean of 350±60 ng/L (Figure 2). The average load for the 24 h period was 17,979±179 mg/h (Figure 3). OC was not recovered (<2.0 ng/L) in the effluent of Benson WWTP during the ‘inter-pandemic’ sampling period, as anticipated.

### Predicted Use of Antiviral from Scenarios

**Figure 4:**
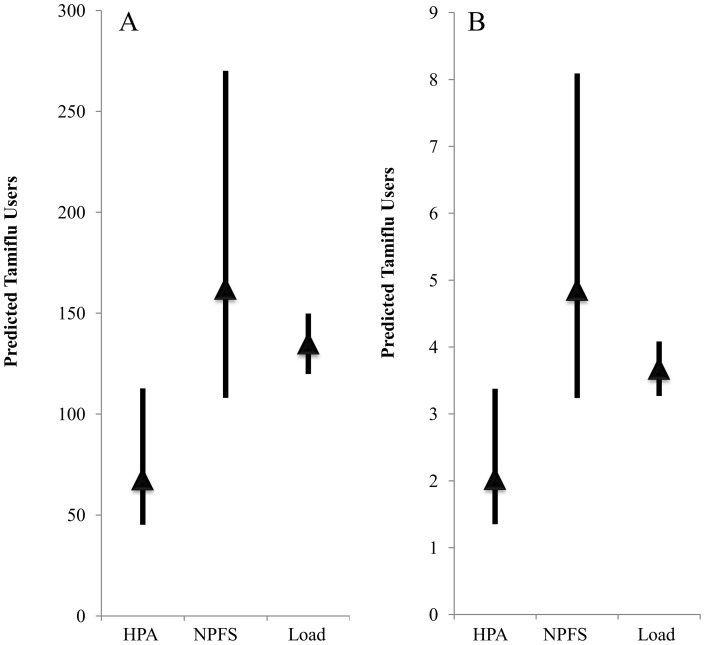
Scenario projections for Tamiflu-consumption. Tamiflu consumption during the 24 hour influent sampling at Oxford (A) and Benson (B) WWTPs based on HPA or NPFS statistics and varying compliance, Tamiflu metabolism, and ILI prescription rates were as detailed in the Materials and Methods. Load represents back-calculated predicted number of Tamiflu users for the 24-h sampling period assuming 80 or 100% OC in the waste stream. The mean of the scenarios is represented by a shaded triangle.

When the full range of compliance (40, 45, 50, 55, 60, 70, 100%) and antiviral allocation rates (50 or 100% of ILI cases) were considered using the HPA population statistics, the Tamiflu-using population was predicted to be between 1.35 and 3.37 people in Benson and 45.1 and 112.7 people in Oxford WWTP catchments (0.02–0.05% of the respective catchment populations; Figure 4).

When the full range of compliance scenarios were considered using the NFPS Tamiflu allocation statistics, the Tamiflu-using population was predicted to be between 3.24 and 8.09 people in Benson and 108 and 270 in Oxford WWTP catchments, respectively (0.05–0.13% of the respective catchment populations; Figure 4).

### Predicted Antiviral released into Waste Water from Scenarios

**Figure 5:**
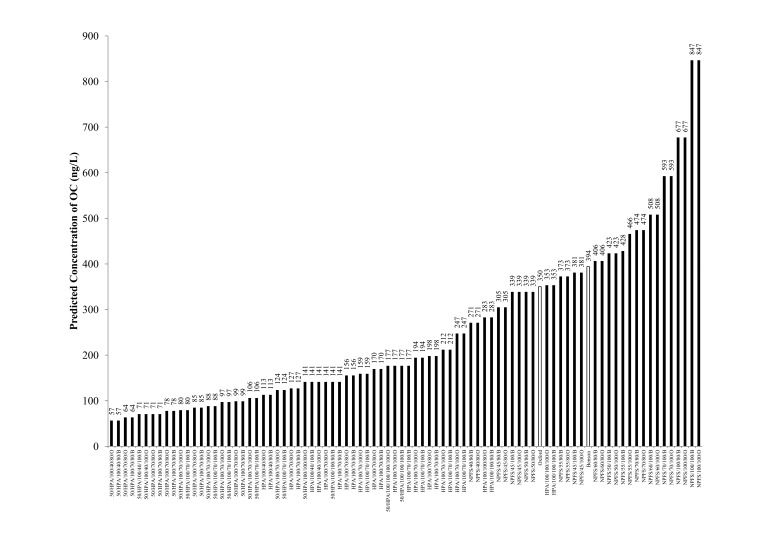
Scenario predictions of OC influent concentration (ng/L). Scenario codes can be interpreted as follows: percent of ILI cases prescribed OC, used only when HPA data was employed (assumed  = 100, unless specified as 50)**/**Source of data (NPFS or HPA)/percent compliance (40, 45, 50, 55, 60, 70, 100%)**/**percent of parent compound converted to OC before WWTP inlet (80, 100%)**/**O =  Oxford WWTP and B  =  Benson WWTP. Mean 24-h OC concentrations at Benson and Oxford are noted by the WWTP name, only. Daily water usage was assumed to be the UK national average of 230 L/capita [3].

Predicted concentrations of OC in the Benson and Oxford WWTP (calculated using Equation 1 including all scenarios discussed in the Material & Methods Section), range from 57 to 847 ng/L (Figure 5). HPA-based scenarios ranged from 57 to 282 ng/L, while NPFS-based scenarios ranged from 270 to 846 ng/L. All the HPA-based scenarios yielded OC concentrations below the measured concentration for Benson (mean 394 ng/L), while only one scenario exceeded the measured concentration for Oxford (mean 350 ng/L). This one scenario was the most conservative, assuming: 100% compliance, 100% of ILI cases were allocated antiviral, and 100% of Tamiflu was recoverable as OC (353 ng/L). Given the unlikely nature of these conservative assumptions, it is argued that the NFPS dataset is a better reflection of antiviral use in the community.

### Predicted Consumption of Antiviral from Measured Load

Measured concentrations of OC were converted to units of Tamiflu users per day. All calculations considered the possibility that the measured concentration of OC reflects either 80 or 100% of the daily dose (0.15 g/d), and in the case of the HPA dataset, it also considered the option that either 50 or 100% of people recorded with ILI were prescribed antiviral. Predicted Tamiflu use from the total 24-h load of OC ranged from 3 to 4 people (0.5–0.6% of the catchment population) in the Benson WWTP catchment and from 120 to 154 people (0.6 to 0.7%) in the Oxford WWTP catchment (Figure 4). Projections remained consistent even when the 24-h mean concentration was used in lieu of load (3 to 4 and 123 to 154 people for Benson and Oxford, respectively).

## Discussion

In this study, Tamiflu use and compliance were predicted from measured OC in waste water influent and through the use of national government statistics on cases of ILI (HPA) and antiviral dispensing (NFPS). Measured concentrations of OC in waste water were consistent with NFPS-derived scenarios. This is not surprising given that NFPS statistics reflect actual antiviral dispensing, albeit at a spatial scale of the UK (62 million), quite dissimilar from the spatial scale of the WWTP (i.e. 6,230 and 240,000 people). HPA-derived scenarios were shown to routinely underestimate Tamiflu use, reflecting the fact that this statistic is derived from regional case rates of ILI determined from patient visits to the general practitioner (GP). Owing to the syndromic diagnosis of ILI during the pandemic via the NPFS website and phone hotline, the HPA-ILI statistic reflects only a fraction of the national ILI-population, as visits to the GP were actively discouraged once the NPFS was fully operational in late-July 2009 [24].

The recovered oseltamivir carboxylate in the Benson and Oxford WWTP catchments during the peak of the autumnal wave in southern England can be best explained by a compliance rate of 45–60% based on NPFS estimates of oseltamivir collection (Figure 5). In a recent study, Singer et al. (2011) modelled Tamiflu use and environmental concentrations of OC during an influenza pandemic of differing severities: R_0_ = 1.65, 1.9 and 2.3, with R_0_ = 1.65 being a good reflection of the 2009 influenza pandemic (R_0_ is the basic reproductive number reflecting the number of cases one case generates on average over the course of their infectious period). Extrapolating from Singer et al. (2011), a pandemic of R_0_ = 1.65 was projected to generate one Tamiflu user within the Benson WWTP catchment and 168 people within the Oxford WWTP catchment on the day of the peak of the pandemic. The model scenario assumes negligible antiviral prophylaxis and the provision of antivirals for treatment of 30% of those with access to antivirals [3]. Projections by Singer et al (2011) were entirely consistent with national antiviral allocation statistics and estimates generated in this study using the waste water epidemiology approach.

The variability in load and concentration of OC in the Benson WWTP influent (relative standard deviation (RSD)  = 132 and 110%, respectively) was much greater than that of Oxford (RSD  = 23 and 17%, respectively). This variability is, to a large extent, a function of the difference in population between Benson and Oxford ( 6230 and 208,000 respectively). Assuming, on average, a person flushes the toilet five times a day [30], the number of flushes per user of Tamiflu (i.e., doses ×5) in Benson ranges from 6.5 to 40 and 225 to 1350 in Oxford (based on NPFS estimates). The low number of flushes per day in Benson and for some scenarios in Oxford would make it difficult to get a representative sampling of Tamiflu users in these catchments using hourly time proportional sampling. A theoretical threshold of 1000 flush events per chemical within a WWTP catchment was proposed as a guide for the minimum flush events needed to justify the use of a time-proportional hourly sampling frequency [30]. Catchments with fewer flush events per day might require more frequent sampling to ensure measured analytes were representative of the pharmaceutical use habits of the catchment population. However, the pumped nature of both of these waste water systems contributes to the mixing of discrete flushing events, particularly important in the smaller Benson catchment during off-peak flow periods. We argue this mixing in the waste water system has alleviated some of the variability associated with sampling small populations at an hourly time interval. However, future studies should give suitable consideration towards minimising the confounding effects of the population size on the waste water sampling.

Antiviral compliance during the 2009 Influenza A(H1N1)pdm09 pandemic has been estimated in a few countries worldwide, most of which were assessed during the early phase of the pandemic on a very small demographic. A study in England examined the degree to which 11–12 yr old pupils of a secondary school complied with a 10-day prophylaxis (once-daily) dosing regimen of Tamiflu at the outset of the pandemic in the UK (April 29, 2009) [4]. The authors found compliance was very high, with 77% taking the full course of Tamiflu [4]. A considerably lower compliance rate of 48% was estimated in a subsequent study that also investigated pupils of a similar age (14 yr) at a boarding school [5]. An online survey of pupils from one primary and two secondary schools in London at the outset of the pandemic in the UK reported only 48% of primary schoolchildren completed a full prophylaxis course, compared to 76% of secondary schoolchildren [6]. A study of compliance in 1–11 yr olds within a nursery, primary school and afterschool club in Scotland reported 97% of the children completed the full prophylaxis regime [7]. The authors proposed that the high compliance might have been related to the socioeconomic status of the population under investigation. Fifty-three (adult) staff and 273 pupils (7–12 yrs old) at a primary school in Sheffield, England were provided Tamiflu for prophylaxis during the latter part of the first wave of the pandemic in the UK (June, 2009) [8]. Of this group, 84% of the pupils and 80% of the staff completed the course of antivirals. It is clear that the survey approach will always be biased towards a demographic and limited in it's scope. In an effort to address these limitations, (web-based) surveys have been implemented during or shortly after the pandemic (e.g., Flusurvey [31], [32]) to fill the knowledge gap. However, such approaches are still biased and thus must be balanced with other sources of information. Here we present the sampling of waste water treatment plant influent as an unbiased method for the determination of drug use and compliance. This study represents the only published report of non-survey based oseltamivir compliance globally and the first report on oseltamivir compliance for the second wave of the pandemic in the UK. Estimated compliance from this study is consistent with the lower range of published compliance rates. The integrated sampling from waste water is proposed as a better measure of compliance as compared to surveys, as this study incorporates all age groups in an unbiased manner, while survey-based studies typically focused on a limited sample size and narrow demographic (<14 year olds).

The accuracy of a waste water epidemiology model for determining drug use and compliance is potentially confounded by inappropriate drug disposal into the waste water itself. In most cases, the active drug that is consumed is excreted into waste water over a period of several hours. In the case of inappropriate drug disposal, the entire dose enters the waste water at once. Presumably when one disposes of drugs in this manner, it entails the disposal of multiple doses at once. Such a bolus of drug might appear realistic if only one waste water sample was taken owing to potential uncertainties with respect to compliance, biodegradation and prescription rates. However, the bolus would appear completely unfeasible with sufficiently high sampling frequency as the quantity of drug passing per unit time would, in comparison to other time points, be unachievable on a per capita basis [30], [33]. Additional supporting evidence for the origin of OC in waste water can come from measuring the ratio of OC:OP, as described in a number of previous studies [17], [34], [35]. However, as OC (the active antiviral) is generated from the prodrug oseltamivir phosphate (OP), it can be assumed that all OC found in waste water in this study was the result of the consumption and (*in vivo*) metabolism of the prodrug.

In summary, we propose waste water forensics can be a valuable tool in monitoring population behaviour and a valuable resource for public health planning. Insight into the proportion of the population that does not utilise allocated antiviral or is not compliant with the dosing regimen could help to inform the development and prioritization of public health provisioning during an influenza pandemic. Owing to the fact that the unit of measurement is a WWTP catchment, the public health message can be targeted and focused towards a particular demographic, with potentially greater efficacy and cost savings. More resolved statistics on the provision of antivirals at the level of the WWTP catchment (e.g., Primary Care Trust) would further improve the power of the model. The forensic epidemiological approach employed in this study might also be applicable to other pharmaceuticals that are highly conserved in the waste stream for which compliance rates are in question.
